# Primary spleen extranodal NK/T cell lymphoma, nasal type, with bone marrow involvement and CD30 positive expression: a case report and literature review

**DOI:** 10.1186/s13000-014-0169-9

**Published:** 2014-09-03

**Authors:** Qinghua Cao, Yan Huang, Ziyin Ye, Ni Liu, Shuhua Li, Tingsheng Peng

**Affiliations:** Department of Pathology, The First Affiliated Hospital of Sun Yat-sen University, 58, Zhongshan Road II, Guangzhou, 510080 China; Department of Pathology, The Sixth Affiliated Hospital of Sun Yat-sen University, 26, Yuancun Erheng Road, Guangzhou, 510655 China

## Abstract

**Aims:**

Primay spleen NK/T cell lymphoma is very rare. We report a case of 39-years-old male of primary splenic NK/T cell lymphoma with bone marrow involvement and CD30 positive expression.

**Case description:**

The patient had high fever for 2 months, and CT scan revealed a diffuse splenomegaly without hepatomegaly. The diagnosis was established by splenectomy specimen and bone marrow biopsy. Normal spleen structure was destroyed by the diffusely infiltrated neoplastic cells, and one of the splenic hilar lymph nodes was involved. The lymphomatous cells were mainly medium-sized, mixed with small and large cells with pleomorphic nuclei and conspicuous nucleoli. Angiocentric growth pattern was present, with mitotic figures and apoptotic bodies easily being found. These neoplastic cells demonstrated a typical immunophenotype of CD2, CD3ε, CD7, CD4, CD56, TIA-1, Granzyme B, CD30 positive, and CD5, CD8, CD20, CD79a negative. The Epstein-Barr virus encoded RNAs (EBERs) genomes were also found in tumor cells by in situ hybridization, while no clonal rearrangement of the T cell receptor-γ genes (TCRG) was found. Biopsy of bone marrow revealed scattered atypical cells presented with a predominantly intrasinusoidal distribution. A diagnosis as primary spleen NK/T cell lymphoma, nasal type (ENKTL) with CD30 expression and bone marrow involvement was finally made. The patient received chemotherapy and was still alive 6 months after splenectomy.

**Clinical significance:**

Primary spleen ENKTL is very rare, it should be made with the combination of clinical feature, PET-CT image, and pathological characteristics, and should be distinguished from other lymphomas or leukemia involved in spleen.

**Virtual Slides:**

The virtual slide(s) for this article can be found here: http://www.diagnosticpathology.diagnomx.eu/vs/13000_2014_169

## Background

Extranodal NK/T cell lymphoma, nasal type, is an uncommon tumor that occurs with a higher prevalence among Asians, and the native American population of Mexico, Central America and South America [1]. It is well known as an aggressive tumor that is characterized by angiocentric and angiodestructive growth pattern, coagulative necrosis mixed with apoptotic bodies, cytotoxic phenotyhpe, and associated with Epstein-Barr Virus (EBV). The most common site of involvement is upper aerodigestive tract (nasal cavity, nasopharynx, paranasal sinuses, palate). Preferential sites of extranasal involvement include the skin, soft tissue, gastrointestinal tract, and testis [1-3]. Rare sites of involvement such as prostate [4], pancreas [5] and adrenal glands [6] have been reported. Primary spleen NK/T cell lymphoma is very rare. Herein, we report a case of primary spleen extranodal NK/T cell lymphoma, nasal type, with bone marrow involvement and CD30 expression.

## Case presentation

### Clinical history

**Figure 1:**
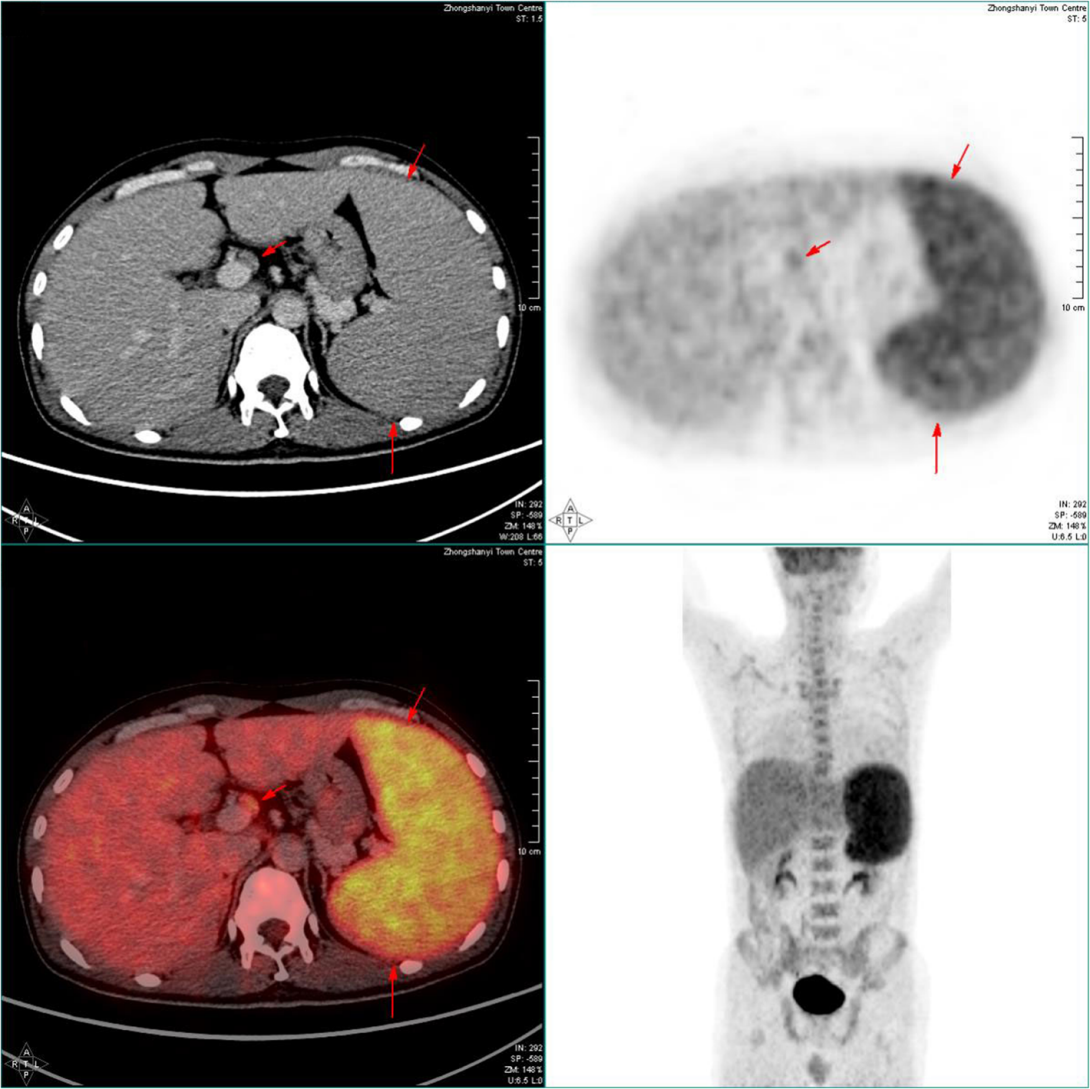
**PET-CT images: FDG PET/CT scan showed the patient had marked splenomegaly (long arrow) and hepatic portal lymphadenopathy (short arrow) with increased FDG metabolism.** The SUV value of the spleen reached 7.2, and that of the hepatic portal lymph node was 2.8, respectively.

A 39-year-old man presented a 2-month history of discontinuous fever peaked up to 39°C without obvious cause, and no response to antibiotic. An ultrasonography and a computed tomography (CT) showed a diffuse splenomegaly without a concrete mass, and lymphadenopathy and hepatomegaly was not detected. FDG PET/CT scan showed significant splenomegaly (long arrow) and hepatic portal lymphadenopathy (short arrow) with increased FDG metabolism (Figure 1). The SUV value reached 7.2 and 2.8 respectively. He denied symptoms of cough, expectoration, erythrism and abdominal pain. Endoscope failed to find lesion in nasal cavity.

Laboratory data showed a leukocyte count of 3.63 × 10^9^/L, haemoglobin 160 g/L, platelet count 208 × 10^9^/L after admission. Bone marrow smear found 2% atypical cells, which was not detected in peripheral blood smear. Unfortunately, the count of platelet decreased quickly to 20 × 10^9^/L within ten days, strongly indicating that the patient had suffered from a highly aggressive tumor in the bone marrow.

Bone marrow biopsy specimen was obtained firstly. A few large atypical cells with T cell immunophenotype were found with intrasinusoidal distributive manner, which provide the clue of the possibility for bone marrow involvement of T/NK tumor cells. For the purpose as diagnostic therapy, the patient received spenoectomy. As the final pathologic diagnosis was spenic NK/T cell lymphoma, nasal type, the patient received five circle chemotherapy afterwards. The patient is alive without recurrence 6 months after the splenectomy.

## Material and methods

The specimen was fixed in a 10% neutral formalin solution and embedded in paraffin. Four micrometer-thick sections were prepared and stained with hematoxylin-eosin. An Envision two-step assay was used for the immunohistochemistry staining. Commercially available monoclonal antibodies were employed CD20 (Mouse mAb(L26);1:200), CD79a (Mouse mAb (JCB117);1:200), CD2 (Mouse mAb (AB75);1:200), CD3ε (Mouse mAb (F7.2.38);1:200), CD5 (Mouse mAb(CD5/54/F6);1:200), CD7 (Mouse mAb(CBC.37);1:200), CD4 (Mouse mAb(4B12);1:200), CD8 (Mouse mAb(C8/144B);1:200), CD56 (Mouse mAb(123C3);1:200), Granzyme B (Mouse mAb(GrB-7);1:200), CD30 (Mouse mAb(Ber-H2);1:200), ALK (Mouse mAb(ALK1);1:200), MPO(Rabbit pAb;1:200),Ki-67 (Mouse mAb(MIB-1);1:200). All the above primary antibodies and HRP-conjugated secondary antibodies were obtained from DAKO Inc., Denmark. The primary antibodies of TdT(Mouse mAb(SEIV28);1:200), TIA-1(Mouse mAb(2G9A10F5);1:200) were from Zhongshan company. The primary antibody of CD123(Mouse mAb(BR4MS);1:400)was from Novocastra.

In situ hybridization (ISH) with Epstein-Barr virus (EBV)-encoded small RNA (EBER) oligonucleotides was performed to test the specimens for the presence of EBV small RNA in formalin-fixed, paraffin-embedded sections using a hybridization kit (DAKO).

For cytogenetic analysis, the paraffin tissue DNA was prepared with a tissue DNA extraction and purification kit (Dneasy TM Tissue Kit, Qiagene, CA). T-cell receptor rearrangement studies were performed. The detection process was conducted by methods described previously [7].

### Pathological findings

**Figure 2:**
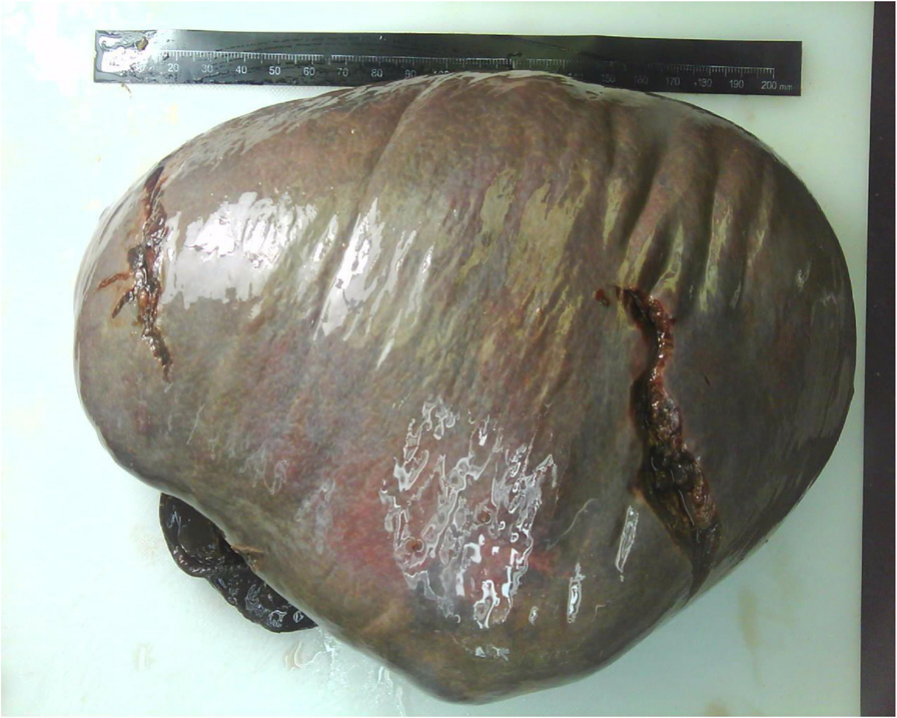
**Macroscopic appearance of the significantly enlarged 18 cm × 15 cm × 10 cm spleen.** Surface and cut surface showed diffuse graywish crimson without nodules.

Grossly, the splenectomy specimen measured 18 cm × 15 cm × 10 cm. Surface and cut surface showed diffuse graywish crimson without nodules. Hemorrhage and necrosis were absent (Figure 2).

**Figure 3:**
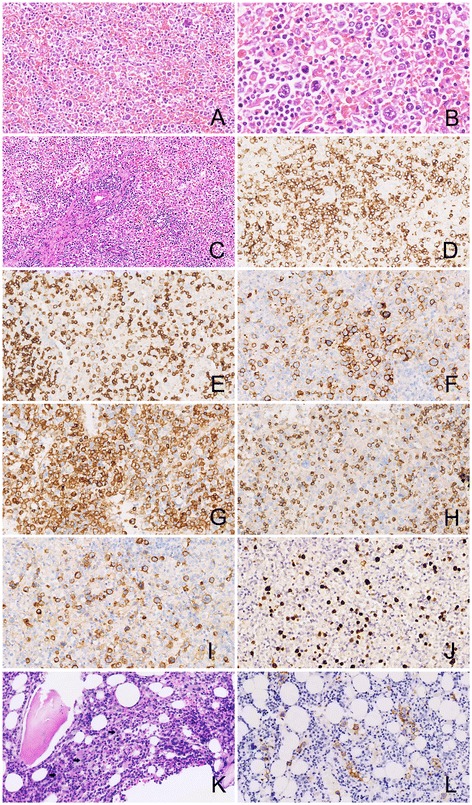
**Microscopic features of the spleen tumor and the bone marrow with immunohistochemical and in situ hybridization staining. (A)** The structure of spleen white and red pulp was destroyed by the diffusely infiltrating neoplastic cells (HE × 100), **(B)** The size of the neoplastic cells were from medium to large with irregular pleomorphic hyperchromatin nuclei and conspicuous nucleoli. Giant tumor cells and apoptotic bodies were easily observed (HE × 200); **(C)** A splenic artery was infiltrated and destroyed by the lymphomatous cells (HE × 40); Tumor cells were postitive for CD2**(D)**, CD3ε **(E)**, CD56 **(F)**, CD4 **(G)**, CD8 **(H)**, and CD30 **(I)** (Envision × 100); **(J)** Tumor cells were positive for EBERs by in situ hybridization; **(K)** Bone marrow biopsy showed scattered medium-sized tumor cells(arrow) (HE × 100); **(L)** Tumor cells of bone marrow were positive for CD56 (Envision × 100).

Microscopically, the structure of normal spleen white pulp and red pulp was destroyed by the diffusely infiltrated neoplastic cells. The lymphomatous cells were mainly medium-sized, mixed with small and large cells, containing moderate pale cytoplasm. Most of them had irregular folded hyperchromatin nuclei and small inconspicuous nucleoli, while the large tumor cells had vesicular pleomorphic nuclei and conspicuous nucleoli (Figure 3A, 3B). Mitotic figures and apoptotic bodies were easily found. The tumor cells were accompanied with an admixture of inflammatory cells as small lymphocytes, plasma cells, and so forth. Many splenic arteries were infiltrated and destroyed by the neoplastic cells (Figure 3C), and geographic coagulative necrosis were also observed. Hemophagocytosis could be found throughout the tumor. The section from the peripheral spleen remained the structure of white pulp and red pulp with expanded sinuses. Neoplastic cells were also found in the cords and sinuses of the expanded red pulp, as well as around splenic arteriolar sheath.

Immunohistochemically, the neoplastic cells were CD2+ (Figure 3D), CD3ε + (Figure 3E), CD7+, CD56+ (Figure 3F), CD4+ (Figure 3G), Granzyme B+, TIA-1+, CD20-, CD79a-, CD5-, CD8- (Figure 3H), ALK-, TdT-, MPO-, and CD123-. Specially, most of the neoplastic cells were CD30 positive (Figure 3I). The proliferation index was approximately 90%, assessed by Ki-67 staining. In situ hybridization (ISH) for EBERs study showed strong positive signals in most of the abnormal large and giant cells (Figure 3J). Based on these results, the diagnosis as spleen exnodular NK/T cell lymphoma, nasal type, with CD30 expression was made. No clonal rearrangement of the T cell receptor gamma (TCRG) genes was found in the lesion by Polymerase Chain Reaction heteroduplex analysis (PCR-HA) and polyacrylamide gel electrophoresis (PAGE).

In addition, five splenic hilar lymph nodes from 0.3 cm to 0.5 cm and one hepatic portal lymph node as 2.8 cm were detected. One of the splenic hilar lymph nodes was involved by the lymphomatous cells with morphology and size similar to which infiltrated into the spleen, while there was lymphoid tissue hyperplasia rather than tumor involvement in the hepatic portal lymph node. The microscopic findings of the bone marrow biopsy revealed scattered atypical cells presented with a predominantly intrasinusoidal distribution. The atypical cells were also medium to large sized, with pale cytoplasm and pleomorphic irregularly folded nuclei (Figure 3K). The scattered atypical cells were stained immunohistochemically as CD2, cytoplasmic CD3ε, CD7, CD56 (Figure 3L), CD30 positive, while CD5, CD20, CD79a, MPO negative. At the same time, EBERs signals were detected in nuclei of the atypical cells. Based on these morphological and immunohistochemical features, it was considered that the bone marrow was involved by the lymphomatous cells of the spleen ENKTL.

## Discussion

According to Brox A [8], the diagnosis of primary lymphoma of the spleen (PSL) should be limited to involvement of only the spleen and splenic hilum. PSL is very rare, which is much less common than the secondary involvement of spleen, accounting for less than 1% of all lymphomas [9]. PSL usually represents non-Hodgkin lymphoma of B cell origin. Shimizu-Kohno K et al. classified 184 specimens of the spleen, 115 were determined to be lymphoid neoplasm (62.5%). The most common subtype of lymphoid neoplasm was diffuse large B-cell lymphoma (DLBCL) (46 cases), followed by splenic marginal zone lymphoma (SMZL) (28 cases), follicular lymphoma (FL) (11 cases), splenic B-cell lymphoma, unclassifiable (SBL-U) (6 cases) and peripheral T-cell lymphoma, not otherwise specified (4 cases, nearly 3.5%) [10]. Hence, T/NK cell lymphoma primarily occurring in spleen is very rare. To our knowledge, this is the first reported case of a primary spleen extranodal NK/T-cell lymphoma, nasal type. The symptoms of the patient were fever, hypocytosis, and splenomegaly, without hepatomegaly and peripheral lymphadenopathy. The neoplastic cells destroyed normal spleen structure, showed typical angiocentric and angiodestructive growth pattern, notable atypical cell morphology, a typical immunophenotype expression of CD56, CD2, CD3ε, and EBERs positive detection with ISH. Additionally, One of the splenic hilar lymph nodes was involved by the lymphomatous cells similar to which infiltrated into the spleen. In this manner, the primary extranodal NK/T cell lymphoma of spleen was confirmed. The bone marrow biopsy revealed scattered atypical cells with NK/T cell immunophenotype and EBERs signals, which highlight the diagnosis as the spleen ENKTL with bone marrow involvement.

The cell morphology of ENKTL is variety. Most cases comprise middle cells mixed a few small- and large-sized cells, and usually do not have nucleoli. In this case the tumor was composed of small to large or anaplastic cells containing several nucleoli. This tissue change may indicate a poor prognosis [1]. And CD30, an important marker for Hodgkin’s lymphoma and Anaplastic large cell lymphoma, is not rarely expressed in ENKTL, which is usually focal and not so intense. Moreover, it was speculated that ENKTL with large cells were prone to express CD30 and indicate a worse prognosis [4,11,12]. On the other hand, there were inverse views as that ENKTL patients with CD30 expression even tended to have more favorable outcome [13], or there was no difference of survival according to CD30 expression [14]. As for the diverse outcome, further study with large number of patients should be performed to evaluated the prognostic value of CD30 expression in NK/T-cell lymphoma. Since there were many CD30 postive lymphomatous cells in this case, the target therapy with Brentuximab (anti-CD30 antibody) might be useful for the clinical trial [15].

ENKTL should be distinguished from other lymphomas or leukemia involved in spleen. Except for the notable atypical cell morphology, ENKTL neoplastic cells have typical immunophenotype expression as CD56, CD2, CD3ε, but usually CD5 negative, and exceptionally CD20 positive [16]. Epstein-Barr virus (EBV) expression is one of the most important factors, and it has been proved that latent membrane protein (LMP) 1 and LMP2A encoded by EBV are also associated with prognostic indicators of survival in patients with ENKTL [17].

The most important differential diagnosis is Aggressive NK-cell leukemia (ANKL), which has been described in a case with jaundice and spontaneous splenic rupture by Gao et al. [18] recently. The immunophenotype of ANKL is identical to that of ENKTL neoplastic cells, and ANKL has been suggested to represent the leukaemic manifestation of ENKTL [1]. But ANKL has a younger median age by more than a decade, and high frequency of hepatosplenic and BM involvement, tumor cells have monomorphic and medium size with irregular nuclei. A fulminant clinical course frequently complicated by multi-organ failure, coagulopathy and haemophagocytic syndrome, and the median survival is less than 2 months. In this case, the patient had only splenomegaly without hepatomegaly, the morphology of neoplastic cells were various, and only a few tumor cells had involved the bone marrow. The clinical course also supported the diagnosis as ENKTL because the patient were still alive for 6 month after the splenectomy and chemotherapy. According to WHO (2008 version), other disorders of NK cell system as Blastic NK-cell lymphoma (BNKL) is also named as Blastic plasmacytoid dendritic cell neoplasm. While actually, there are still difference between “real” NK-cell lymphoma and Blastic plasmacytoid dendritic cell neoplasm, because of the morphology of the tumor cells, the sites of involvement, the immunophonotype and association with EBV [1].

Hepatosplenic T-cell lymphoma (HSTL) is another rare T-cell lymphoma needing to be distinguished. HSTL cells usually derive from a functionally immature cytotoxic γδ T cell,and usually occurs in adolescents and young adults(68%) [19]. Patients initially present with fever, fatigue, weight loss, and abdominal discomfort due to splenomegaly and hepatomegaly. The neoplastic cells involve the cords and sinuses of the splenic red pulp, with a reduction or complete atrophy of the white pulp. Hepatomegaly can be found in approximately 50% of the patients, and the liver also shows a predominant sinusoidal infiltration. The cells of HSTL are monotonous, with medium-sized nuclei and a rim of pale cytoplasm. The neoplastic cells are CD3+, and usually TCRδ1+, TCRαβ-, CD56+/−,CD4-,CD8−/+,TIA-1+,GranzymeB-, and EBV is generally negative. Cases of γδ origin show a biallelic rearrangement of TRG genes, and TRB genes are arranged in αβ cases [1]. In this case, all of the features including the tumor morphology, immunophenotype, EBERs positive expression, and TCRG negatively rearrangement did not support the diagnosis as HSTL.

Since some of the tumor cells were large and anaplastic with CD30 positive staining, Anaplastic large cell lymphoma (ALCL) should also be distinguished. All cases of ALCL contain a variable proportion of “hallmark” cells with eccentric, horseshoe- or kidney-shaped nuclei often with an eosinophilic region near the nucleus. The tumor cells are positive for CD30 on the cell membrane and in the Golgi region, and usually ALK positive. ALCL are also consistently negative for EBV (EBER or LMP1) [1]. In this case, the morphology of the neoplastic cell were different from ALCL, and the tumor cells were ALK- and EBER+. Hence, the diagnosis of ALCL in spleen should be excluded.

Spleen and peripheral lymph nodes can also be affected by Peripheral T-cell lymphoma, NOS (PTCL,NOS). The PTCL neoplastic cells are medium-sized and/or large cells with irregular, pleomorphic, hyperchromatic or vesicular nuclei, prominent nucleoli and many mitotic figures. The lymphomatous cells are usually T-cell phenotype positive with downregulation of CD5 and CD7, TCR βF1 positive and EBV negative. PTCL, NOS is a diagnosis with the exclusion of other specific T/NK cell lymphoma [1]. In this case, the tumor cells’ morphology, immunophenotype and EBER positive improve the diagnosis as an ENKTL.

The rearrangement TCR genes is an important supplement to the diagnosis of T-cell non-Hodgkin lymphoma. TCR genes are clonally rearranged in most cases of PTCL, NOS [20], while only a small proportion of ENKTL show clonal rearrangement [21,22]. According to all of the characteristics of the tumors’ morphology, immunophenotype, EBERs ISH, and TCR rearrangement in this case, other type of lymphomas as Hodgkin lymphoma (HL) and diffuse large B cell lymphoma (DLBCL) could be ruled out from the differential diagnosis.

## Conclusions

The case reported a rare primary NK/T cell lymphoma in spleen. As spleen can be involved by other types of lymphomas, the final diagnosis should be made with the combination of clinical feature, PET-CT image, pathological morphology, immunophenotype, and genetic features. The prognosis of those tumors comprised of big cells with CD30 positive expression are quite unclear. As agrressive NK cell leukemia shared similar morphology and immunophenotype features with ENKTL, sites of involvement and clinical course may serve as important clues to make the distinction between them.

## Consent

Written informed consent was obtained from the patient for publication of this Case Report and any accompanying images. A copy of the written consent is available for review by the Editor-in-Chief of this journal.

## Abbreviations

PSL: Primary spleen lymphoma
ANCL: Aggressive NK cell leukemia
ALCL: Anaplastic large-cell lymphomas
HL: Hodgkin lymphoma
DLBCL: Diffuse large B cell lymphoma
SMZL: Splenic marginal zone lymphoma
FL: Follicular lymphoma
